# Passive Aggression and Life Satisfaction in Middle Adulthood: Sequential Mediating Roles of Emotion-Regulation Difficulties and Interpersonal Relationship Quality

**DOI:** 10.3390/bs16081272

**Published:** 2026-07-24

**Authors:** Young-Ok Lim, Jae-Sun An, Kunho Lee, Kyung-Hyun Suh

**Affiliations:** Department of Counseling Psychology, Sahmyook University, Seoul 01795, Republic of Korea; yvonneok@hanmail.net (Y.-O.L.); lucky603@syu.ac.kr (J.-S.A.); leekunho@syu.ac.kr (K.L.)

**Keywords:** passive aggression, emotion-regulation, interpersonal relationship, life satisfaction

## Abstract

This study examined the relationship between passive aggression and life satisfaction among middle-aged Korean adults, focusing on the sequential mediating roles of emotion-regulation difficulties and interpersonal relationship quality. Participants were 299 adults aged 40–64 years who completed an online survey. The proposed sequential mediation model was tested using PROCESS Macro 4.2 Model 6 with 5000 bootstrap samples. Passive aggression was positively associated with emotion-regulation difficulties and negatively associated with interpersonal relationship quality and life satisfaction. Emotion-regulation difficulties were negatively associated with both the quality of interpersonal relationship and life satisfaction, whereas interpersonal relationship quality was positively associated with life satisfaction. Sequential mediation analysis indicated that passive aggression did not directly predict life satisfaction when the mediators were included in the model. Instead, the indirect effects through emotion-regulation difficulties and interpersonal relationship quality were statistically significant, whereas the remaining direct effect of passive aggression on life satisfaction was not significant. These findings were consistent with a pathway in which passive aggression was associated with greater emotion-regulation difficulties, poorer interpersonal relationship quality, and lower life satisfaction.

## 1. Introduction

When individuals experience anger or hostility toward others, they may act aggressively (Berkout et al., 2019). However, because overt aggression can damage interpersonal relationships and lead to negative social or legal consequences, some individuals’ express hostility in more indirect ways (Yesavage, 1983). Passive aggression refers to a pattern of indirect, non-confrontational behaviors through which individuals express negative emotions or resist demands while avoiding direct conflict (Long et al., 2009). Although passive-aggressive behaviors are less overt than direct aggression, they can impair interpersonal relationships and are associated with depression, psychological maladjustment, and self-destructive tendencies (Schanz et al., 2022). Therefore, passive aggression may be harmful to both oneself and others.

Individuals exhibiting pronounced passive-aggressive tendencies were previously considered to have a distinct personality disorder. Although passive-aggressive personality disorder (PAPD) was included in earlier editions of the Diagnostic and Statistical Manual of Mental Disorders (DSM), it was gradually deemphasized and ultimately removed from DSM-5 (American Psychiatric Association, 1987, 2013). Similarly, PAPD is no longer recognized as a distinct diagnostic category in the current International Classification of Diseases (ICD-11; World Health Organization, 2020).

Despite its removal from contemporary diagnostic systems, passive-aggressive personality features continue to attract clinical and research attention. Previous studies have shown that PAPD frequently co-occurs with other personality disorders and psychiatric conditions, indicating that passive-aggressive traits reflect a clinically meaningful pattern of maladaptive personality functioning rather than an obsolete diagnostic construct (Becker et al., 2000; Czajkowski et al., 2008). Furthermore, passive-aggressive tendencies have been associated with various psychological difficulties, including depression, interpersonal dysfunction, and broader psychological maladjustment (Czajkowski et al., 2008; Millon, 1981; Schanz et al., 2022). These findings suggest that, although PAPD is no longer recognized as a formal diagnosis, passive-aggressive tendencies remain an important construct for understanding mental health and interpersonal functioning.

While few studies examine the relationship between passive aggression and life satisfaction, existing evidence suggests that passive-aggressive tendencies may adversely affect an individual’s well-being by negatively impacting interpersonal functioning. Passive-aggressive tendencies have been associated with heightened interpersonal distress, particularly vindictive and spiteful interpersonal problems (Winer et al., 2024). Individuals with stronger passive-aggressive tendencies often experience recurring relational conflicts characterized by resentment, covert resistance, and antagonistic attitudes, which undermine the quality and stability of interpersonal relationships (Laverdière et al., 2019). Moreover, passive-aggressive tendencies may interfere with effective problem-solving in social situations, further impairing relationship functioning (Gál et al., 2025).

Given that high-quality interpersonal relationships are a key contributor to subjective well-being and life satisfaction (Asiri, 2019; Rojas, 2026; Whitton et al., 2014; Zhang et al., 2024), individuals with strong passive-aggressive tendencies may be at greater risk of lower life satisfaction because of difficulties in their interpersonal relationships. Because such tendencies are characterized by interpersonal conflict and problematic social interactions (Gál et al., 2025; Laverdière et al., 2019; Winer et al., 2024), they are likely to undermine relationship quality and in turn, reduce life satisfaction. Therefore, this study examined the relationship between passive aggression and life satisfaction and proposed that passive aggression is associated with life satisfaction through its detrimental effects on interpersonal relationship quality.

The proposed model is grounded in an integrated emotion-regulation and interpersonal-process framework. The multidimensional perspective on emotion regulation suggests that difficulties in understanding, accepting, and managing negative emotions may be associated with maladaptive expressions of anger and hostility (Gratz & Roemer, 2004). From an interpersonal-process perspective, indirect hostility, avoidance, and covert resistance may interfere with open communication and constructive conflict resolution, thereby undermining relationship quality (Long et al., 2009; Laverdière et al., 2019). Because interpersonal relationship quality is closely associated with life satisfaction (Whitton et al., 2014; Zhang et al., 2024), these perspectives together provide a theoretical rationale for a sequential pathway linking passive aggression, emotion-regulation difficulties, interpersonal relationship quality, and life satisfaction.

Moreover, this study investigated the psychological mechanisms by which passive aggression may be associated with poorer interpersonal relationship quality. Previous research has consistently demonstrated that aggression is associated with emotion-regulation difficulties (Donahue et al., 2014; Garofalo et al., 2018; Lin et al., 2024), and passive-aggressive tendencies are positively related to difficulties in emotion-regulation (Al-Shafey, 2023). Furthermore, emotion-regulation difficulties are associated with greater interpersonal conflicts and poorer relationship functioning, whereas effective emotion-regulation contributes to healthier interpersonal relationships (Haliczer et al., 2021; Malkoç et al., 2019). Consistent with the integrated framework described above, we proposed that passive aggression would be associated with greater emotion-regulation difficulties, which would in turn be associated with poorer interpersonal relationship quality and lower life satisfaction. Emotion-regulation difficulties were positioned before interpersonal relationship quality because problems in managing negative emotions are expected to influence communication, responses to conflict, and relationship maintenance, whereas interpersonal relationship quality reflects the accumulated consequences of these regulatory difficulties.

Despite these findings, several gaps remain in the literature. First, although passive-aggressive tendencies have been linked to psychological maladjustment and interpersonal dysfunction, their association with global life satisfaction has rarely been examined directly. Second, previous studies have generally examined aggression and emotion-regulation difficulties, passive aggression and interpersonal dysfunction, or interpersonal relationships and life satisfaction separately (Donahue et al., 2014; Laverdière et al., 2019; Whitton et al., 2014), leaving unclear how these processes jointly account for life satisfaction. In particular, the sequential pathway from passive aggression to emotion-regulation difficulties, poorer interpersonal relationship quality, and lower life satisfaction has not been adequately examined. This mechanism also remains understudied in middle adulthood, when emotional regulation and interpersonal relationships are closely tied to well-being. The present study addresses these gaps by testing an integrated sequential mediation model among middle-aged Korean adults.

Therefore, this study examined the relationships among passive aggression, emotion-regulation difficulties, interpersonal relationship quality, and life satisfaction in middle-aged Korean adults as well as the sequential mediating roles of emotion-regulation difficulties and interpersonal relationship quality on the association between passive aggression and life satisfaction. Accordingly, the following hypotheses were proposed:

**H1.**  *Passive aggression, emotion-regulation difficulties, interpersonal relationship quality, and life satisfaction are significantly associated with one another among middle-aged Korean adults.*

**H2.**  *Emotion-regulation difficulties and interpersonal relationship quality sequentially mediate the relationship between passive aggression and life satisfaction.*

**Figure 1:**
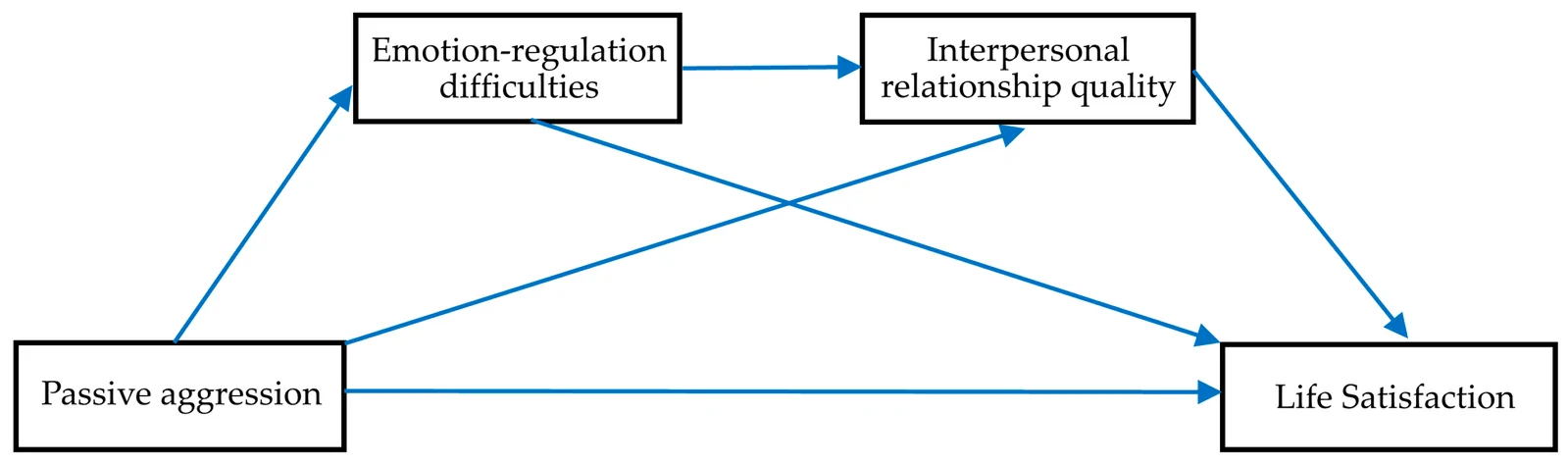
Double sequential mediation model proposed in this study.

The hypothesized model is illustrated in Figure 1.

## 2. Materials and Methods

### 2.1. Participants

The sample consisted of 299 middle-aged Korean adults (men = 133 [44.5%], women = 166 [55.5%]) aged 40–64 years. With respect to age, 93 participants (31.1%) were in their 40s, 116 (38.8%) were in their 50s, and 90 (30.1%) were in their 60s. Most participants (*n* = 268, 89.6%) were married or living with a spouse, whereas 31 (10.4%) were single, divorced, or widowed. In addition, 277 participants (92.6%) reported having one or more children.

### 2.2. Data Collection

Data were collected via an online survey using Google Forms from June 20 to July 9, 2025. Participants were recruited through social media platforms and the researchers’ personal social networks. Eligibility criteria were being a Korean adult aged 40–64 years and providing electronic informed consent. Although no formal quota sampling was applied, efforts were made to obtain a balanced distribution of gender and age groups. Google Forms required a response to every item before participants could proceed to the next page or submit the questionnaire. Consequently, no incomplete responses were submitted, and all 299 responses were included in the final analysis. No responses were excluded on the basis of duplicate submission, unusually rapid completion, or patterned responding because no prespecified screening thresholds for these criteria had been established. A formal a priori power analysis was not conducted; however, the final sample of 299 participants was considered sufficient for estimating the proposed regression-based mediation model using bootstrap confidence intervals.

This study was approved by the Institutional Review Board (IRB No. 2025-05-035-001). Electronic informed consent was obtained before participation, and participants were informed of their right to withdraw from the study at any time without penalty.

### 2.3. Instruments

#### 2.3.1. Passive Aggression

Passive-aggressive tendencies were measured using the Passive Aggression Scale (PAS) developed and validated by Lim and Suh (2022). The PAS is a 21-item self-report instrument designed to assess passive-aggressive behaviors among Korean adults. It comprises three subscales: inducing criticism, which reflects behaviors intended to elicit negative evaluations or disapproval from others; avoiding and ignoring, which assesses tendencies to evade communication or disregard others as an indirect expression of hostility; and sabotaging, which refers to covert behaviors that obstruct or undermine others’ goals or activities. All items were rated on a six-point Likert scale ranging from 1 (not at all true) to 6 (very true), with higher scores indicating greater passive-aggressive tendencies. In the present study, only the total scores were used in the analyses, and the scale demonstrated excellent internal consistency with a Cronbach’s α of 0.91.

#### 2.3.2. Emotion-Regulation Difficulties

Emotion-regulation difficulties were assessed using the Korean version of the Difficulties in Emotion-Regulation Scale, which was adapted and validated by Cho (2007) based on the original scale developed by Gratz and Roemer (2004). The instrument consists of 35 items that measure six facets of emotion-regulation difficulties: non-acceptance of emotional responses, issues with goal-directed behavior, impulse control, lack of emotional awareness, limited access to effective emotion-regulation strategies, and lack of emotional clarity. Responses were recorded on a five-point Likert scale ranging from 1 (almost never) to 5 (almost always), with higher scores indicating greater difficulties in emotion-regulation. In the present study, only the total score was used in the analyses, and the scale demonstrated excellent internal consistency, with a Cronbach’s α of 0.94.

#### 2.3.3. Interpersonal Relationship Quality

Interpersonal relationship quality was measured using the Relationship Change Scale, originally developed by Schein and Guerney (1975) and adapted for use in the Korean population by Moon (1980). This scale is designed to assess the overall quality of interpersonal relationships and consists of 25 items across seven dimensions: satisfaction, communication, trust, intimacy, sensitivity, openness, and understanding. Responses are rated on a five-point Likert scale, with higher scores indicating better interpersonal relationship quality. In the present study, only the total score was used in the analyses, and the scale demonstrated excellent internal consistency, with a Cronbach’s α of 0.93.

#### 2.3.4. Life Satisfaction

Life satisfaction was assessed using the Korean version of the Satisfaction with Life Scale (SWLS), originally developed by Diener et al. (1985) and translated and validated by Kim (2007). The SWLS is a widely used measure designed to assess individuals’ global cognitive evaluations of their overall quality of life. The scale consists of five items rated on a seven-point Likert scale ranging from 1 (strongly disagree) to 7 (strongly agree), with higher scores indicating greater life satisfaction. Total scores range from 5 to 35. In the present study, only the total score was used in the analyses, and the scale demonstrated excellent internal consistency, with a Cronbach’s α of 0.90.

### 2.4. Statistical Analysis

Data analyses were performed using IBM SPSS Statistics for Windows, Version 25.0 (IBM Corp., Armonk, NY, USA) and PROCESS Macro version 4.2. Before the main analyses, the assumption of normality was evaluated by examining the skewness and kurtosis of the study variables. Mean item scores, rather than summed scores, were used for all four variables in the correlation and mediation analyses. Accordingly, the unstandardized regression coefficients are expressed in mean-item-score units. Pearson’s correlation analyses were conducted to investigate the relationships among the variables. To test the proposed sequential mediation model, PROCESS Macro Model 6 (Hayes, 2018) was employed. The significance of the indirect effects was assessed using a bootstrap procedure with 5000 resamples and 95% bias-corrected (BC) confidence intervals (CIs).

## 3. Results

### 3.1. Relationship Among the Study Variables Involved in Life Satisfaction

Table 1 shows the correlations among passive aggression, emotion-regulation difficulties, interpersonal relationship quality, and life satisfaction of middle-aged Korean adults. Because the absolute values of skewness and kurtosis for all variables were less than 1, the data were deemed to approximate a normal distribution. Accordingly, the assumption of normality was satisfied, justifying the application of parametric statistical procedures (Kline, 2005).

Correlational analysis revealed that passive aggression was positively correlated with emotion-regulation difficulties (*r* = 0.336, *p* < 0.001) and negatively correlated with interpersonal relationship quality (*r* = −0.264, *p* < 0.001) and life satisfaction (*r* = −0.150, *p* < 0.01). Emotion-regulation difficulties were negatively correlated with interpersonal relationship quality (*r* = −0.472, *p* < 0.001) and life satisfaction (*r* = −0.411, *p* < 0.001). In contrast, interpersonal relationship quality was positively correlated with life satisfaction *(r* = 0.593, *p* < 0.001).

### 3.2. Verification of the Double Mediation Model for Life Satisfaction

This study examined the sequential double mediating effects of emotion-regulation difficulties and interpersonal relationship quality on the association between passive aggression and life satisfaction among middle-aged Korean adults (Table 2). Before conducting the main analyses, multicollinearity among the predictor variables was assessed. Based on the criteria proposed by West et al. (1995), tolerance values below 0.20 and variance inflation factor (VIF) values exceeding 5.0 suggest problematic multicollinearity. In the present study, tolerance values ranged from 0.729 to 0.873, and VIF values ranged from 1.146 to 1.371, indicating that multicollinearity was not a concern.

The results indicated that passive aggression positively predicted emotion-regulation difficulties (*B* = 0.217, *p* < 0.001). In the model predicting interpersonal relationship quality, both passive aggression and emotion-regulation difficulties were negative predictors (*B* = −0.068, *p* < 0.05; *B* = −0.380, *p* < 0.001, respectively). In the final model predicting life satisfaction, passive aggression did not significantly predict life satisfaction (*B* = 0.069, *p* = 0.324). However, emotion-regulation difficulties negatively predicted life satisfaction (*B* = −0.396, *p* < 0.001), whereas interpersonal relationship quality positively predicted life satisfaction (*B* = 1.289, *p* < 0.001).

**Figure 2:**
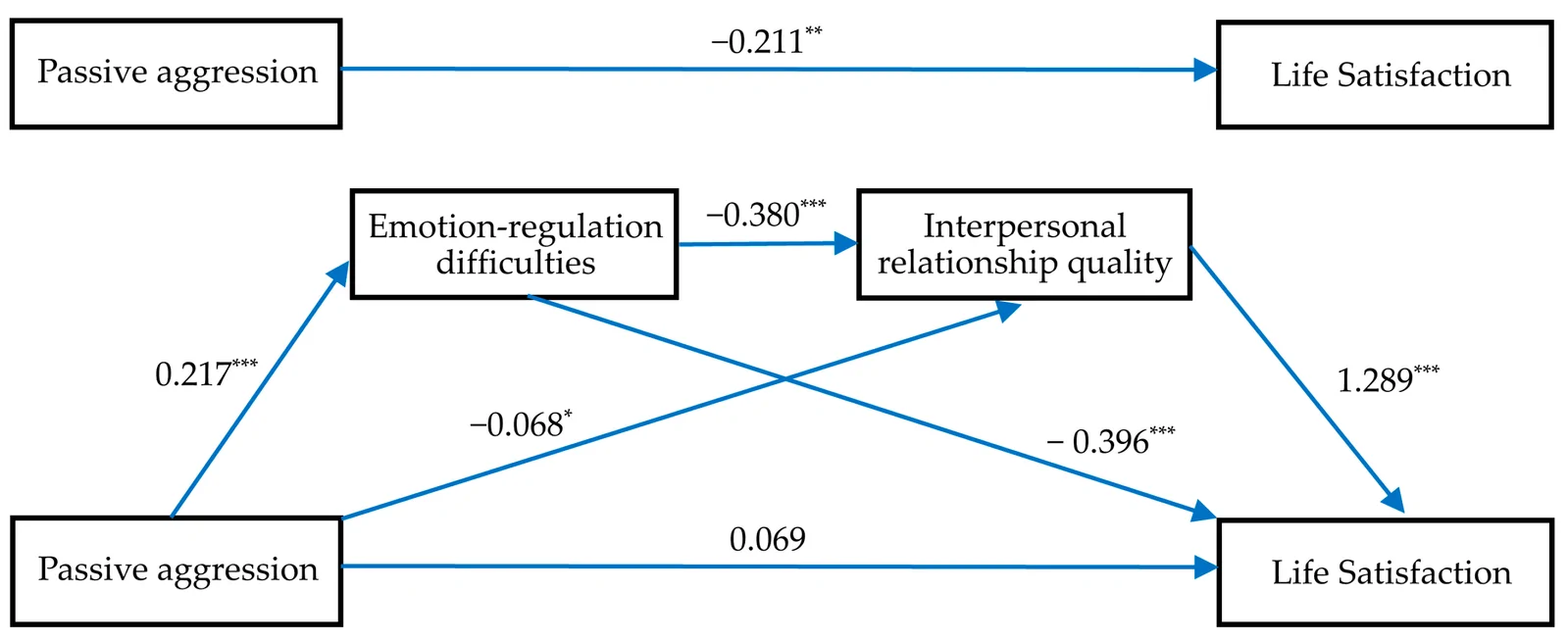
Examined double mediation model of emotion-regulation difficulties and interpersonal relationship quality on passive aggression and life satisfaction (* *p* < 0.05, ** *p* < 0.01, *** *p* < 0.001; Unstandardized coefficients).

As shown in Figure 2, passive aggression significantly predicted life satisfaction in the total-effects model (*B* = −0.211, *p* < 0.01). However, when emotion-regulation difficulties and interpersonal relationship quality were included as sequential mediators, the direct effect of passive aggression on life satisfaction was no longer significant. Notably, the coefficient for the direct effect changed from negative in the total-effect model to positive, although nonsignificant, after the mediators were included (*B* = 0.069).

As shown in Table 3, the bootstrap analysis based on 5000 resamples revealed a significant total indirect effect of passive aggression on life satisfaction (Effect = −0.280, 95% BC CI [−0.3994, −0.1726]). Additionally, all specific indirect effects were statistically significant because their 95% CIs did not include zero. The indirect effects through emotion-regulation difficulties (A → B → D; Effect = −0.086, 95% BC CI [−0.1463, −0.0375]), interpersonal relationship quality (A → C → D; Effect = −0.087, 95% BC CI [−0.1807, −0.0026]), and the sequential pathway involving emotion-regulation difficulties and interpersonal relationship quality (A → B → C → D; Effect = −0.107, 95% BC CI [−0.1672, −0.0587]) were all significant. These findings indicate significant specific and total indirect effects consistent with the proposed sequential pathway.

## 4. Discussion

This study investigated the relationships among passive aggression, emotion-regulation difficulties, interpersonal relationship quality, and life satisfaction in middle-aged Korean adults. Additionally, we examined the sequential mediating roles of emotion-regulation difficulties and interpersonal relationship quality in the association between passive aggression and life satisfaction. The findings and implications of this study are discussed below.

We found that passive aggression was negatively associated with life satisfaction among middle-aged Korean adults, although this association was relatively modest. The magnitude of this association was small (*r* = −0.150), indicating that passive aggression alone was only weakly related to life satisfaction. This finding suggests that individuals with strong passive-aggressive tendencies tend to evaluate their lives less positively. One possible explanation is that passive aggression is characterized by chronic resentment, indirect hostility, and difficulties in managing interpersonal conflicts (Long et al., 2009), which may contribute to interpersonal stress and reduced well-being. Such difficulties may be particularly important during middle adulthood, when maintaining stable family, work, and social relationships is closely related to life satisfaction. This small association suggests that passive aggression may not be a strong independent determinant of life satisfaction, but may be more relevant when considered together with emotional and interpersonal difficulties.

Another possible explanation is that passive aggression is associated with broader psychological maladjustment and poor mental health. Previous studies have linked passive-aggressive tendencies to depression and personality dysfunction (Czajkowski et al., 2008; Schanz et al., 2022), while individuals with mental health difficulties generally report lower life satisfaction (Lombardo et al., 2018; Meule & Voderholzer, 2020). Thus, the negative association observed in this study may reflect a link between passive aggression, psychological distress, and reduced well-being.

Another finding of this study is that passive aggression was negatively associated with interpersonal relationship quality among middle-aged Korean adults. This pattern is consistent with previous research suggesting that passive-aggressive tendencies are characterized by resentment, covert resistance, and difficulties in resolving interpersonal conflicts, which may undermine relationship quality (Gál et al., 2025; Laverdière et al., 2019). Individuals with strong passive-aggressive tendencies often avoid direct communication and express dissatisfaction indirectly, and these tendencies may be accompanied by greater misunderstanding, lower trust, and greater relational tension. Accordingly, stronger passive-aggressive tendencies may be associated with lower-quality interpersonal relationships. This interpretation is consistent with the possibility that the interpersonal consequences of passive aggression arise less from overt conflict than from repeated indirect behaviors that gradually weaken trust and communication.

This finding may be particularly meaningful in middle adulthood because relationship quality plays a central role in psychological adjustment and well-being during this life stage. By comparison, interpersonal relationship quality showed a relatively strong association with life satisfaction (*r* = 0.593), whereas the associations involving emotion-regulation difficulties were moderate (*r* = −0.411 to −0.472). A recent meta-analysis demonstrated that relationship satisfaction remains an important component of well-being across the lifespan and is closely linked to overall life functioning (Bühler et al., 2021). As passive-aggressive behaviors interfere with open communication and constructive conflict resolution, they may hinder the development and maintenance of satisfying relationships. Therefore, passive aggression may function as a maladaptive interpersonal style that contributes to poor relationship quality among middle-aged adults.

This study also found that passive aggression was positively associated with emotion-regulation difficulties. In addition, emotion-regulation difficulties significantly mediated the relationship between passive aggression and interpersonal relationship quality. These findings are consistent with previous research demonstrating that aggressive tendencies are associated with difficulties in emotion-regulation (Donahue et al., 2014; Garofalo et al., 2018; Lin et al., 2024) and that passive-aggressive tendencies are positively related to emotion-regulation difficulties (Al-Shafey, 2023). Individuals with strong passive-aggressive tendencies may have difficulty regulating negative emotions such as anger, frustration, and resentment. Consequently, they may rely on indirect and maladaptive forms of emotional expression that are characteristic of passive-aggressive behavior.

The mediating role of emotion-regulation difficulties suggests that passive aggression may be associated with poorer interpersonal relationship quality partly through its association with difficulties in emotion regulation. Previous studies have shown that emotion-regulation difficulties are associated with greater interpersonal conflict and poorer relationship functioning, whereas effective emotion-regulation facilitates healthier and satisfying interpersonal relationships (Garofalo et al., 2018; Haliczer et al., 2021; Malkoç et al., 2019). Individuals who struggle to regulate their emotions may be more likely to respond maladaptively to interpersonal stress, which may be associated with less open communication and poorer relationship functioning. Therefore, the present findings suggest that emotion-regulation difficulties may be a mechanism through which passive aggression is associated with poor interpersonal relationship quality among middle-aged adults.

A particularly noteworthy finding of this study is that the indirect effects through emotion-regulation difficulties and interpersonal relationship quality were significant, whereas the remaining direct effect of passive aggression on life satisfaction was not statistically significant. Although passive aggression was significantly associated with lower life satisfaction in the total-effect model, the direct coefficient became positive but remained statistically nonsignificant when the mediators were included. This finding suggests that the association between passive aggression and life satisfaction may be statistically accounted for by its associations with greater emotion-regulation difficulties and poorer interpersonal relationship quality. In other words, stronger passive-aggressive tendencies were associated with greater emotion-regulation difficulties, poorer interpersonal relationships, and lower life satisfaction in a pattern consistent with the proposed sequential pathway. The non-significant direct effect further suggests that the association with life satisfaction may be explained primarily by emotional and relational processes rather than by passive aggression alone. However, because the data were cross-sectional, this interpretation represents a plausible explanatory pathway rather than a confirmed causal sequence. Alternatively, broader psychological distress or other unmeasured personal and contextual factors may contribute simultaneously to passive aggression, emotion-regulation difficulties, poorer relationships, and lower life satisfaction.

These findings highlight the importance of emotional and interpersonal processes in understanding the link between passive aggression and well-being among middle-aged adults. From clinical and community-based intervention perspectives, the results suggest that emotion-regulation skills and interpersonal relationship quality may represent relevant targets for interventions addressing passive-aggressive tendencies and low life satisfaction. Accordingly, programs designed to promote adaptive emotional coping and effective interpersonal communication may warrant further evaluation among middle-aged adults with passive-aggressive tendencies.

This study has several limitations that must be acknowledged. Participants were recruited through an online survey using social media platforms and the researchers’ personal networks, and the sample included slightly more women than men. Because convenience sampling rather than probability sampling was used, the sample may not be representative of middle-aged Korean adults and may be subject to selection bias. Accordingly, the findings should be interpreted with caution, and their generalizability may be limited. And, all variables were assessed using self-report measures, raising the possibility of response bias, and only total scores were analyzed, precluding examination of the unique effects of specific subdimensions. In addition, no control variables were included in the mediation model. Although this decision avoided post hoc adjustment for variables lacking a clear theoretical or causal rationale, the possible influence of unmeasured demographic, psychological, or contextual confounders cannot be ruled out. Future studies should identify and prespecify theoretically relevant covariates and examine whether the proposed associations remain consistent after their inclusion.

Finally, because the study employed a cross-sectional design, causal relationships among passive aggression, emotion-regulation difficulties, interpersonal relationship quality, and life satisfaction cannot be established. Although the ordering of the variables was guided by the proposed theoretical framework, the sequential mediation model does not establish temporal precedence or causal direction. Alternative and bidirectional pathways remain plausible; for example, emotion-regulation difficulties may contribute to passive-aggressive behavior rather than result from it. Longitudinal or experimental studies are needed to compare these competing temporal models. Future research should therefore use longitudinal or experimental designs, recruit more representative samples, incorporate multiple assessment methods, and examine subscale-level relationships.

## 5. Conclusions

This study investigated the relationships among passive aggression, emotion-regulation difficulties, interpersonal relationship quality, and life satisfaction in middle-aged Korean adults. The findings revealed that passive aggression was negatively associated with interpersonal relationship quality and life satisfaction, and positively associated with emotion-regulation difficulties. Furthermore, significant indirect effects were observed through emotion-regulation difficulties and interpersonal relationship quality, whereas the remaining direct effect was not statistically significant. These results were consistent with an indirect statistical pathway linking passive aggression with greater emotion-regulation difficulties, poorer interpersonal relationship quality, and lower life satisfaction. These findings highlight the importance of emotional and interpersonal processes in understanding well-being among middle-aged adults and point to interventions that focus on enhancing emotion-regulation skills and interpersonal relationship quality.

## Figures and Tables

**Table 1:** Correlational matrix of passive aggression, emotion-regulation difficulties, interpersonal relationship quality, and life satisfaction (*N* = 299).

Variables	1	2	3	4
1. Passive aggression	1			
2. Emotion-regulation difficulties	0.336 ***	1		
3. Interpersonal relationship quality	−0.264 ***	−0.472 ***	1	
4. Life satisfaction	−0.150 **	−0.411 ***	0.593 ***	1
*M*	2.49	2.25	3.65	4.73
*SD*	0.82	0.53	0.47	1.16
Skewness	0.38	0.14	−0.01	−0.43
Kurtosis	−0.27	−0.18	0.23	−0.28

** *p* < 0.01, *** *p* < 0.001. *M*, mean; *SD*, standard deviation. All descriptive statistics are based on mean item scores.

**Table 2:** Double mediating effect of emotion-regulation difficulties and interpersonal relationship quality on passive aggression and life satisfaction.

Variables	*B*	*S.E.*	*T*	LLCI	ULCI
Mediating Variable Model (Outcome Variable: Emotion-regulation difficulties)					
Constant	1.709	0.093	18.44 ***	1.5265	1.8913
Passive aggression	0.217	0.035	6.15 ***	0.1477	0.2868
Mediating Variable Model (Outcome Variable: Interpersonal relationship quality)					
Constant	4.677	0.111	42.10 ***	4.4587	4.8960
Passive aggression	−0.068	0.031	−2.21 *	−0.1282	−0.0073
Emotion-regulation difficulties	−0.380	0.048	−8.00 ***	−0.4736	−0.2867
Dependent Variable Model (Outcome Variable: Life satisfaction)					
Constant	0.735	0.658	1.12	−0.5601	2.0307
Passive aggression	0.069	0.069	0.99	−0.0680	0.2053
Emotion-regulation difficulties	−0.396	0.117	−3.37 ***	−0.6270	−0.1647
Interpersonal relationship quality	1.289	0.130	9.89 ***	1.0328	1.5457

* *p* < 0.05, *** *p* < 0.001. *S.E*.: standard error; LLCI: lower level for confidence interval; ULCI: upper level for confidence interval.

**Table 3:** Indirect effects of the mediation model.

Path	Effect	*S.E.*	95% BC CI
Total indirect effect	−0.280	0.058	−0.3994~−0.1726
Ind 1: A → B → D	−0.086	0.028	−0.1463~−0.0375
Ind 2: A → C → D	−0.087	0.045	−0.1807~−0.0026
Ind 3: A → B → C → D	−0.107	0.027	−0.1672~−0.0587

A = Passive aggression, B = emotion-regulation difficulties, C = Interpersonal relationship quality, D = Life satisfaction. *S.E*.: standard error; CI: confidence interval.

## Data Availability

The datasets analyzed in this study are available from the corresponding author upon reasonable request.

## Abbreviations

PAS	Passive Aggression Scale
SWLS	Satisfaction with Life Scale
IRB	institutional review board
SPSS	Statistical Package for Social Sciences
VIF	variance inflation factor
LLCI	lower level for confidence interval
ULCI	upper level for confidence interval

## References

[B1-behavsci-16-01272] Al-Shafey N. F. A. (2023). Passive-aggressive personality disorder and its relation to cognitive biases and emotion regulation difficulties among university students: A psychometric–clinical study. The Educational Journal of the Faculty of Education, Sohag University.

[B2-behavsci-16-01272] American Psychiatric Association (1987). Diagnostic and statistical manual of mental disorders.

[B3-behavsci-16-01272] American Psychiatric Association (2013). Diagnostic and statistical manual of mental disorders.

[B4-behavsci-16-01272] Asiri Y. (2019). Interpersonal relationship and life satisfaction differences between urban and rural environments.

[B5-behavsci-16-01272] Becker D. F., Grilo C. M., Edell W. S., McGlashan T. H. (2000). Comorbidity of borderline personality disorder with other personality disorders in hospitalized adolescents and adults. American Journal of Psychiatry.

[B6-behavsci-16-01272] Berkout O. V., Tinsley D., Flynn M. K. (2019). A review of anger, hostility, and aggression from an ACT perspective. Journal of Contextual Behavioral Science.

[B7-behavsci-16-01272] Bühler J. L., Krauss S., Orth U. (2021). Development of relationship satisfaction across the life span: A systematic review and meta-analysis. Psychological Bulletin.

[B8-behavsci-16-01272] Cho Y. (2007). Assessing emotion dysregulation: Psychometric properties of the Korean version of the Difficulties in Emotion Regulation Scale. Korean Journal of Clinical Psychology.

[B9-behavsci-16-01272] Czajkowski N., Kendler K. S., Jacobson K. C., Tambs K., Røysamb E., Reichborn-Kjennerud T. (2008). Passive-aggressive (negativistic) personality disorder: A population-based twin study. Journal of Personality Disorders.

[B10-behavsci-16-01272] Diener E., Emmons R. A., Larsen R. J., Griffin S. (1985). The satisfaction with life scale. Journal of Personality Assessment.

[B11-behavsci-16-01272] Donahue J. J., Goranson A. C., McClure K. S., Van Male L. M. (2014). Emotion dysregulation, negative affect, and aggression: A moderated, multiple mediator analysis. Personality and Individual Differences.

[B12-behavsci-16-01272] Garofalo C., Velotti P., Zavattini G. C. (2018). Emotion regulation and aggression: The incremental contribution of alexithymia, impulsivity, and emotion dysregulation facets. Psychology of Violence.

[B13-behavsci-16-01272] Gál Z., Nagy M. T., Takács I. K., Kasik L. (2025). The relationship between social problem-solving and passive-aggressive behavior among adolescents. European Journal of Investigation in Health, Psychology and Education.

[B14-behavsci-16-01272] Gratz K. L., Roemer L. (2004). Multidimensional assessment of emotion regulation and dysregulation: Development, factor structure, and initial validation of the difficulties in emotion regulation scale. Journal of Psychopathology and Behavioral Assessment.

[B15-behavsci-16-01272] Haliczer L. A., Woods S. E., Dixon-Gordon K. L. (2021). Emotion regulation difficulties and interpersonal conflict in borderline personality disorder. Personality Disorders: Theory, Research, and Treatment.

[B16-behavsci-16-01272] Hayes A. F. (2018). Introduction to mediation, moderation, and conditional process analysis: A regression-based approach.

[B17-behavsci-16-01272] Kim J. (2007). The relationship between life satisfaction/life satisfaction expectancy and stress/well-being: An application of motivational states theory. Korean Journal of Health Psychology.

[B18-behavsci-16-01272] Kline R. B. (2005). Principles and practice of structural equation modeling.

[B19-behavsci-16-01272] Laverdière O., Ogrodniczuk J. S., Kealy D. (2019). Interpersonal problems associated with passive-aggressive personality disorder. Journal of Nervous and Mental Disease.

[B20-behavsci-16-01272] Lim Y.-O., Suh K.-H. (2022). Development and validation of a measure of passive aggression traits: The passive aggression scale (PAS). Behavioral Sciences.

[B21-behavsci-16-01272] Lin S., Cheng G., Sun S., Feng M., Bai X. (2024). Emotional regulation of displaced aggression in provocative situations among junior high school students. Behavioral Sciences.

[B22-behavsci-16-01272] Lombardo P., Jones W., Wang L., Shen X., Goldner E. M., Bassani D. G. (2018). The fundamental association between mental health and life satisfaction: Results from successive waves of a Canadian national survey. BMC Public Health.

[B23-behavsci-16-01272] Long N. J., Long J. E., Whitson S. (2009). The angry smile: The psychology of passive–aggressive behavior in families, schools, and workplaces.

[B24-behavsci-16-01272] Malkoç A., Aslan Gördesli M., Arslan R., Çekici F., Aydin Sunbul Z. (2019). The relationship between interpersonal emotion regulation and interpersonal competence controlled for emotion dysregulation. International Journal of Higher Education.

[B25-behavsci-16-01272] Meule A., Voderholzer U. (2020). Life satisfaction in persons with mental disorders. Quality of Life Research.

[B26-behavsci-16-01272] Millon T. (1981). Disorders of personality: DSM–III, Axis II.

[B27-behavsci-16-01272] Moon S. M. (1980). A study on the effects of human relations training group counseling. Journal of Gyeongsang University.

[B28-behavsci-16-01272] Rojas M. (2026). Which interpersonal relationships are more important for wellbeing? Evidence from three Latin American countries. The Journal of Development Studies.

[B29-behavsci-16-01272] Schanz C. G., Equit M., Schäfer S. K., Michael T. (2022). Self-directed passive-aggressive behaviour as an essential component of depression: Findings from two cross-sectional observational studies. BMC Psychiatry.

[B30-behavsci-16-01272] Schein S., Guerney B. G. (1975). The interpersonal relationship scale. Unpublished Doctoral dissertation.

[B31-behavsci-16-01272] West S. G., Finch J. F., Curran P. J., Hoyle R. H. (1995). Structural equation models with nonnormal variables: Problems and remedies. Structural equation modeling: Concepts, issues, and applications.

[B32-behavsci-16-01272] Whitton S. W., Rhoades G. K., Whisman M. A. (2014). Fluctuation in relationship quality over time and individual well-being: Main, mediated, and moderated effects. Personality and Social Psychology Bulletin.

[B33-behavsci-16-01272] Winer S., Ramos Salazar L., Anderson A. M., Busch M. (2024). Resolving conflict in interpersonal relationships using passive, aggressive, and assertive verbal statements. International Journal of Conflict Management.

[B34-behavsci-16-01272] World Health Organization (2020). International statistical classification of diseases and related health problems.

[B35-behavsci-16-01272] Yesavage J. A. (1983). Direct and indirect hostility and self-destructive behavior by hospitalized depressives. Acta Psychiatrica Scandinavica.

[B36-behavsci-16-01272] Zhang J., Zhao S., Deng H., Yuan C., Yang Z. (2024). Influence of interpersonal relationship on subjective well-being of college students: The mediating role of psychological capital. PLoS ONE.

